# Open instrumentation, like open data, is key to reproducible science. Yet, without incentives it won’t thrive

**DOI:** 10.1098/rsta.2023.0215

**Published:** 2024-06-03

**Authors:** Julian Stirling

**Affiliations:** ^1^ Freelance researcher, Bath, UK

**Keywords:** open source hardware

## Abstract

There is far more to open science than simply not shutting away your work. For example, a significant time investment must be made to collate, curate, explain and document precisely how an experiment can be reproduced or data can be reused. This time investment is currently poorly rewarded in our current model of open science where a bare minimum of openness is mandated, but further work is not recognized. As the open science movement looks beyond open access publications and open data towards ongoing detailed work such as open source software and open source hardware, it needs to consider how to properly encourage the extra work that is needed to properly document these projects. Without detailed documentation, the work cannot be replicated, reused and continually improved. If the work cannot be replicated or reused, is it really even open?

This article is part of the Theo Murphy meeting issue ‘Open, reproducible hardware for microscopy’.

## 1. Introduction

Experimental science relies on the assumption that experiments reported in the literature can be replicated. Moreover, it relies on the assumption that the experiment will give the same result when replicated. It is becoming clear that these assumptions are not as true as we would hope [1,2]. A few high profile cases may be explained by mistakes that invalidate conclusions [3] or even outright scientific fraud [4]. Yet, most cases might be explained by something much more mundane: our inability to replicate many results is simply the expected outcome for complex experiments where the devil is in the details, but those details remain unpublished.

The movement towards open science should be addressing reproducibility head on. Movements towards requiring open data archives [5], and encouraging open source scientific software [6] theoretically allow researchers to reanalyse and reuse data from the literature. Ideally the raw data, exactly as it is taken from an instrument, should be in this archive. If any custom analysis scripts are provided alongside the raw data, then the analysis can be replicated in full. Ideally, the provided analysis will create the exact figures used in a publication. If we strive to meet this level of openness, we can ensure that data analysis can be replicated, reproduced, reused, examined and (if needed) corrected. The public who fund scientific research are repeatedly told that science is collaborative and self-correcting, and making this sort of data archive the norm would go a long way towards this.

Many groups, however, meet the data availability requirements by exporting their final analysed data in a CSV file. This reduces the data availability requirement to a metaphorical lipstick for pigs. Though who can blame these groups, when they exist within a system that incentivizes this behaviour? Reviewers are not asked to check the data archives to meet the ideals described above. Meeting the ideals above takes time [5]. Meeting the ideals above increases the chance that another group will find an error in your work. Meeting the ideals above gives other groups access to your analysis scripts, speeding up their research. The last point should be seen as a scientific benefit, but it cannot be in a system that ranks researchers on their ability to outpublish their peers.

Data availability and open source scientific software can solve reproducing results from already collected data, yet making the data reproducible requires replication of an experiment. For fields where custom or customized instrumentation are the norm, how is it possible to truly replicate any experiment? Experimental methods and instruments are published in their own specialist journals. Some new journals are encouraging full hardware designs to be published alongside the manuscript [7,8]. However, most instrumentation journals only require scientists to explain the operational principles of the instrument and provide example results. The journals generally do not require enough information to be published to allow another researcher to precisely replicate the instrument. If the instrument cannot be replicated, how can any future experiments using this instrument be replicated?

Slowly, the need for open source science hardware is entering into the wider discussion of open science. Notably, open source hardware forms a key theme in international guidance provided by the UNESCO Recommendation on Open Science [9]. At a national level, the UK Reproducibility Network has released a primer on open source hardware, focusing not only on how to make hardware open source but also on why academics should consider it [10]. Stated benefits to releasing hardware as open source include research integrity, replicability, collaboration and accessibility. Making hardware open source takes considerable time, the noble benefits that are listed above again do not align well within our current systems for ranking research.

## 2. There is more to being open than simply not being closed

A very simplistic view of open science might simply be science where nothing is hidden, allowing anyone to replicate any given result. However, in practice, the information required to truly understand and replicate a specific experiment is not contained in a single digital directory that can be made open at the click of a button. The actual information needed is held within lab books and notes, within the memories of multiple researchers, it exists as physical objects in the laboratory. Even the information that is stored digitally is likely spread over multiple computers, over multiple directories, with custom software relying on the specific configuration of a specific computer. Considerable work is required to collate, archive, document and explain the information to make it truly useful to others.

In the specific case of experiments that require the development of custom instrumentation, there is a wide spectrum of openness. At the less open end of the spectrum, the ability to replicate a specific instrument would be significantly increased if technical drawings of custom components were provided, along with a computer-aided design (CAD) file that provides a three-dimensional model of the final assembled instrument. Yet, this still would require significant work for projects that were assembled exactly once during the ongoing design; often assembled from components that were originally specified by an informal conversation with technical staff rather than from a detailed technical drawing.

To make the instrumentation more open, a researcher can try to release enough information to meet the open source hardware definition. The most common definition of open source hardware is provided by the Open Source Hardware Association (OSHWA). To meet the OSHWA definition for open source hardware [11], all of the editable CAD files should be shared in their original format. By requiring CAD to be shared in its original format, the CAD file will show more than the final shape of the object. The CAD file will also provide step-by-step the design operations that were taken to create this shape. This extra information both helps to provide some of the design intent and makes it easier for others to continue the design. Even with every original design file for an instrument, true replication requires further information. DIN, the German Institute for Standardization, has begun the process of formalizing an open source hardware definition that lays out the information that is required for different types of hardware to be considered open source [12]. For example, the assembly and calibration procedures play a big part in the performance of a scientific instrument; this information should be provided for the instrument to be considered open source. However, if an instrument is a one-off custom design, the assembly and calibration procedures as explained will likely rely heavily on the skill, experience and intuition of the researcher performing the assembly and calibration.

The most ambitious open source hardware projects try to build a community that designs the instrument in collaboration. The open source hardware movement is strongly modelled on the open source software movement. Famous projects like the Linux operating system (which now powers most Internet servers, most supercomputers and most mobile phones) are written by thousands of developers across the world [13]. It is clear to everyone involved that the operating system will never be complete, it will always be improving and developing. This continuous ongoing open development model, if replicated for science hardware, could enable the best researchers in the world to collaborate on instrumentation. If scientists could build new innovations directly on top of state-of-the-art instruments without first having to recreate the base instruments they are improving, this could materially accelerate scientific innovation. However, this open collaboration model takes considerable work.

On the most open end of the spectrum, the OpenFlexure project [14] is a scientific hardware project that has attempted to apply this open source development model to hardware [15]. The entire project is developed entirely using open source software, so that no collaborator is locked out by not having the correct licenses. Development discussions, project management, version history and experimental adaptations are all hosted in the open. As part of the project, we have put significant effort into adding procedures for approving major changes written up formally as OpenFlexure Enhancement Proposals, as well as less onerous procedures for signing off any changes that enter the main ongoing development version of the microscope. This focus on open process and open source software has led the project to develop numerous associated open source software projects [16], and to spend considerable time on developing and sharing its workflows [17]. This has created a network of many hundreds of users who have independently reproduced the microscope and led to the development of numerous other microscopes built on top of the same base system [18,19]. As a researcher involved with this project for many years, it is hard not to ask whether I would have had better career progression if I had chosen to chase academic statistics rather than an idealistic standard for open research.

## 3. The lack of incentives for openness and interoperability

The direction of academic research is driven predominantly by grant funding and the academic prestige associated with countable statistics, such as citations, h-index, etc. Academia’s focus on bibliometrics creates a zero sum game where researchers are continuously in competition, incentivized to exploit knowledge for publications rather than share it freely for the benefit of the community. Looking back through historical literature before these academic pressures, it is hard not to conclude that our current push for open science is not a newfangled revolution, but a return to a Victorian view of science that is building communal knowledge. For example in Joule’s pioneering work on the expansion of gases in 1845, he writes:

I must here be permitted to make a short digression, in order to explain the construction of the stop-cocks, as it may save those who may in future attempt similar experiments, the useless trouble of trying to make the ordinary stop-cock perfectly air-tight under high pressures. The one I have used is the invention of Mr. Ash, of this town, a gentleman well known for his great mechanical genius; and he has in the most obliging manner allowed me to give a full description of it. Fig. 4 is a full-sized sectional view of the stop-cock. [20]

Joule then continues to provide a clear description of the stop-cock’s construction which takes up most of a page. Would most modern academic journals allow (let alone encourage) such a long description of an incidental mechanical component simply because it will aid replication of the experiment? If not, this represents a significant regression in our commitment to reproducible science.

A key theme of the recent Royal Society meeting on ‘Open, reproducible hardware for microscopy’ was the need for standardization of both mechanical components and software interfaces to allow instruments to be adapted, maintained, repaired and improved more easily. This problem of non-standardized components in machinery is in my experience a key theme in discussions of open source hardware in general. This ongoing discussion of component standardization dates back to the Victorian era and to the first industrial revolution. Joseph Whitworth, a pioneer of precision engineering, delivered an impassioned paper to the Institution of Civil Engineers in 1841 which began:

The screw threads which form the subject of this paper are those of bolts and screws, used in fitting up steam engines and other machinery. Great inconvenience is found to arise from the variety of threads adopted by different manufacturers. The general provision for repairs is rendered at once expensive and imperfect. The difficulty of ascertaining the exact pitch of a particular thread, especially when it is not a multiple or submultiple of the common inch measure, occasions extreme embarrassment. This evil would be completely obviated by uniformity of system, the thread becoming constant for a given diameter. [21]

Just 18 years after Whitworth’s first suggestion that screw threads should be standardized, Richard Beck suggested in the 1859 Transactions of the Microscopical Society that a similar Whitworth screw profile with a 0.8 inch major diameter and 36 threads to the inch should be adopted as a standard for microscope objectives. This thread was indeed adopted, and then adjusted and further formalized as The Royal Microscopical Society’s (RMS) standard screw-thread for objectives in 1896 [22]. This RMS objective thread standard remains the dominant thread standard, forming part of ISO 9345:2019 [23], 160 years after it was first introduced.

Microscopy’s early introduction of such an important standard must have contributed to the ability of microscopists to collaborate and improve their instrumentation. While it is certainly impressive that this core standard has remained dominant for so long, it is concerning that over a century and a half later, the microscopy community is still struggling with a lack of standardized components.

Twenty-first century laboratories are not without standardized interfaces. Instruments for the past decades have communicated through a combination of USB, GPIB or Serial buses; with many instruments fitting standardized 19 inch racking. Not to mention, the standardized mechanical components that are commonplace in the twenty-first century. However, these standards have developed within industry not within academia. When you consider more specialist hardware only seen in academic laboratories, interoperability tends to decrease. It is worth questioning why academic instrument development has not developed more interoperable interfaces over the past century. One reason must surely be that the low volumes of such instruments being sold to any institution gives academics very little power to influence instrument manufacturers. However, in my opinion, it is perverse incentives within academia itself that drives the lack of interoperability.

Consider a new instrument, or instrument interface, designed within academia. As discussed previously, the work to fully describe and document the instrument well enough that it can be replicated, and interfaced with other instrumentation, is significant. This work is unlikely to form part of an academic paper, and therefore is unlikely to generate citations. The academic impact of such documentation may well be huge over time (as the longevity of the standardized RMS thread shows), but it would not be an impact that would be simple to track and present for academic career development. Instead, the pressure within academia is to patent new instrumentation and to create a spin-out company. A patent grants the university, the spin-out or any downstream purchaser of the patent a monopoly on the instrument, and thus any other manufacturer creating interoperable components now faces the potential of a lawsuit.

The prevalence of patents within academia should itself be considered. The ostensible economic justification for the creation of the patent was to speed innovation by encouraging inventors to explain their innovations openly. The reward for this openness is a time limited monopoly to help them fund their innovations [24]. Even as early as the Victorian era, there was a strong pushback against patents as they impeded innovation rather than accelerated it. Isambard Kingdom Brunel, often considered to be Britain’s most prolific engineer, famously announced in 1851 that:

I have never taken out a Patent myself, or ever thought of doing so and I have gradually become convinced that the whole system of Patents … is one productive of immense evil. [25]

It is concerning that the chief engineer employed by ninteenth century railway entrepreneurs was free to avoid the monopolies that patents provide, whereas twenty-first century academic contracts can be used to bind researchers to seek these same monopolies. The question that should be asked is how the economic argument for patents can possibly apply to academia? Academic funding is not driven from the proceeds of exploiting these monopolies, it is generally driven by public funding. The patent does not incentivize open sharing of innovation within academia, instead, it delays publication while patent proceedings are ongoing. As such, no part of the economic arguments for patents applies to academic development of scientific instruments.

Both the OSHWA and DIN definitions of open source hardware require licenses that enshrine the right for the hardware to be made and sold, even by for-profit entities. Thus, any release of instrumentation as open source hardware does not preclude commercialization of the research. Requiring the open source release of hardware developed in academia can achieve the desired goal of speeding innovation through open explanation, without the delays and monopolies of the patent system.

## 4. Conclusion

Experimental science remains a key pillar of science in the digital age. The ability to physically test a theory or hypothesis must always be part of science. As such, the movement for open science cannot overlook the experiments themselves, and the instruments that enable these experiments. For open science to truly thrive, we must support open sharing of experimental setups including the full and open descriptions of any custom instrumentation. However, this requirement for open sharing cannot simply be bolted on as an afterthought without considering the considerable time investment required to share this knowledge well enough for it to be useful. Current academic evaluations based on patents or high-profile research papers reward results that are initially seen as novel or unexpected, yet this does not mean the work is reproducible [26]. Monitoring citations tells you whether this work was considered noteworthy by others, but no metric in common use captures whether the results were actually replicated or directly used by others.

Open science is currently mandated rather than incentivized. The use of sticks rather than carrots may seem more assertive to funding bodies that require specific information to be open. However, this creates hoops for academics to jump through, proving only that an exact minimum requirement is achieved rather than providing incentives for going up and beyond. In the future, if funding bodies truly want to encourage open science, they must work with universities to develop incentives that aid career progression rather than rely on mandating a minimum benchmark. Open source hardware as a new and upcoming field is still developing its best practices. Funding bodies and institutions should be helping to develop these best practices to ensure that they improve the reproducibility of experiments and that good practice can be tracked and incentivized [27]. If these incentives are designed well enough, modern open science might be able to rekindle the open spirit of Victorian science.

## Data Availability

This article has no additional data.
